# Effectiveness of Percutaneous Intradiscal Decompression Therapy in Thoracic Disc Herniation

**DOI:** 10.4274/balkanmedj.galenos.2018.2018.0188

**Published:** 2019-02-28

**Authors:** Ayşegül Ceylan, Güngör Enver Özgencil, Burak Erken, İbrahim Aşık

**Affiliations:** 1 Department of Anesthesiology and Reanimation, University of Health Sciences, Gülhane Training and Research Hospital, Ankara, Turkey; 2 Department of Anesthesiology and Reanimation, Ankara University Faculty of Medicine Hospital, Ankara, Turkey

**Keywords:** Intervertebral disc disease, pain, percutaneous decompression, thoracic disc herniation

## Abstract

**Aims:**

Although there have been many studies about lumbar and cervical ablation procedures, few studies have been performed in the thoracic region. To evaluate the clinical results of a percutaneous disc decompression device in patients with radicular symptoms and/or dorsal pain due to thoracic disc herniation.

**Methods:**

Eleven patients with thoracic disc herniation and/or degenerative discs (all in T10-T11, or T11-T12 levels) who did not respond to conservative treatments were undergoing ablation and compression procedures. Pain and radicular symptoms consistent with the thoracolumbar region were confirmed via abnormal magnetic resonance imaging findings after detailed anamnesis and physical examination. All patients were evaluated before and 1, 3, 6, and 12 months after treatment using the visual analog scale score. The patient satisfaction scale was used to evaluate the level of patient satisfaction at the end of the treatment at 12 months.

**Results:**

The median visual analog scale score was 7.00±0.45 points before treatment and 2.73±0.65 points at 12 months post-procedure and were statistically significant (p<0.001). The results of pairwise comparisons using the Bonferroni Corrected Wilcoxon Signed-Rank test showed that there were statistically significant differences. The mean visual analog scale score at the beginning (7.00±0.45) was significantly higher than the mean score of other months. Postoperative improvement was significant with a 99% confidence interval. No complications that may cause permanent damage occurred.

**Conclusion:**

Percutaneous disc decompression is an effective and safe procedure to treat pain caused by lower thoracic intervertebral disc disease, which did not respond to conservative treatments.

Thoracic disc herniation is a rare disease with an incidence of 1/1,000,000 per year (1). However, its incidence and prevalence associated with radiculopathy and/or myelopathy is unknown. Thoracic disc herniation is responsible for 2% of patients with back pain. Approximately 0.15-1.8% of all thoracic disc herniation patients are treated surgically (2). In the vast majority of patients, trauma is often an initiating factor. It is a story of an accidental axial loading such as falling on the hips, a prolonged posture of flexion, incorrect posture, or heavy lifting. Afflicted individuals are initially asymptomatic and may eventually become symptomatic with physical activity (3). Certain impact injuries, such as parachute landings, can cause damage to the thoracic discs. Invasive treatment usually involves a thoracotomy procedure with discectomy or fusion implantation. Commonly, symptoms of thoracic disc hernias may be poorly defined and involve pain in the back, waist, and legs, numbness, coldness, or loss of strength (4) that are not well localized. Symptoms are twice as likely to occur bilaterally. However, both sides may not be affected equally. Discogenic pain may heal incompletely or relapse without alleviating causative factors such as instable vertebral segments, carrying heavy loads, and hard working conditions.

In 75% of thoracic disc herniation patients, the affected level is below T8 and is mostly at T11-T12 (5). This predominance may be because the lower thoracic level is more mobile and the posterior longitudinal ligament at this level is relatively weaker when compared with other levels. Thoracic disc herniation is more centrally located and more likely to be calcified than both cervical and lumbar disc hernias (6).

Patients with thoracic disc herniation may have a wide variety of symptoms; the most common is continuous or intermittent back pain which is usually described as burning or stabbing (7,8). Depending on the location of the lesion, pain may also be as side pain, abdominal pain, or groin pain. It is not uncommon for patients to be accidentally diagnosed as cholelithiasis, nephrolithiasis, or gastritis (9). A detailed physical/neurological examination and radiological imaging should be performed in patients who are suspected of thoracic disc herniation. In patients without myelopathy, it is possible to miss thoracic pathology and to focus on the lumbar degenerative condition. Asking for the location of the pain will help to make a correct diagnosis.

Recently, a routable/navigable percutaneous decompression instrument called L’DISQ^TM^ (U&I Co. Ltd., Uijeongbu, Korea) has been introduced to treat degenerative discs. It has a wheel-shaped knob that can be rotated to provide the desired angle and be controlled within the disc and has a conductive electrode accessible to the back tear ring of the desired region (10,11,12). The plasma energy produced at the distal end of the bar evaporates the nucleus pulposus and ablates nearby soft tissue. The direct removal of hernia tissues by L’DISQ^TM^ provides the user with a proposed advantage by permitting entrance into larger hernias by extruding fragments, which are now regarded as contraindications for many percutaneous instruments (13,14).

Despite studies about lumbar and cervical ablation procedures, few studies have been performed in the thoracic region. To evaluate the clinical results of percutaneous disc decompression (L’DISQ^TM^) applications in patients with lower radicular symptoms and/or dorsal pain due to thoracic disc herniation who did not respond to conservative methods.

## MATERIALS AND METHODS

After approval by the Ethics Committee, we retrospectively screened the data of 11 patients who were treated with a percutaneous thoracic disc ablation technique using L’DISQ^TM^ device in the algology clinic between 2013 and 2017. All data including anamnesis, physical examinations, plain radiography, magnetic resonance imaging, routine blood tests, and bleeding profile results were evaluated.

### Patient selection

Inclusion criteria were being older than 18 years and in American Society of Anesthesiologists I-II risk groups, having a visual analog scale score >4 points, having resistant symptoms despite facet joint blockade, thoracic epidural steroid injection, physical therapy, muscle relaxant, or anti-inflammatory treatments for at least 3 months.

**Figure 1:**
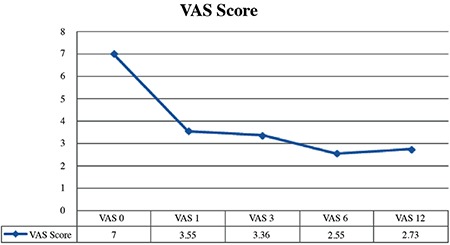
Visual analog scale scores. VAS: visual analog scale

Pain and clinical symptoms of patients, which were detected by anamnesis and neurological examinations, were compatible with magnetic resonance imaging and patient characteristics and magnetic resonance imaging findings (Table 1, Figure 1).

Exclusion criteria were patients with root compression or zygapophyseal arthrosis shown on plain X-ray and lumbar magnetic resonance imaging, vertebral fracture, previous thoracic spine surgery, signs or symptoms of thoracic canal stenosis, psychological disorder, localized or systemic infection, tumors, coagulopathy, pregnancy, osteoarthritis, and marked disc degeneration. Patients in American Society of Anesthesiologists ≥III risk group were also excluded because of potential comorbidities that might increase the complication rate for outpatient procedures.

### Procedure

All patients had previously failed facet injections or medial branch blocks. All procedures were performed by a single experienced algologist. During the procedure, blood pressure, electrocardiography, oxygen saturation were monitored.

The procedures were performed under sterile conditions and fluoroscopy using a standard oblique intradiscal approach. Before the procedure, all patients were administered intravenous antibiotic cefazolin, 1 g for prophylaxis, and midazolam 2-5 mg for reducing anxiety and discomfort. Then, the patients became calm but alert and conscious, so that they could talk to the practitioner in case of unusual pain.

Both the angle of vertebral entrance of the L’DISQ^TM^ cannula and the distance from the skin entrance point to the midline are different in lumbar and thoracic regions. The lateral approach is standard at the L1-L4 level (15). A far posterolateral approach with an entrance 12-14 cm away from the vertebral midline and an angle of 45 degrees is preferred at the L5-S1 level, whereas an angle of 60 degrees and a distance of 3-4 cm from the vertebrae are optimal for the thoracic level. The mode, duration, or power of the device is not changed during thoracic or lumbar applications. It is different for cervical vertebrae. A new device called L’DISQ-C^TM^ was developed, which contains an electrode with a shorter and smaller diameter tip than the lumbar catheter that facilitates the entry into the narrow cervical intervertebral disc region (16).

After taking the prone position on the operating table, the operational area was cleaned, and covered with sterile cloths, and an interventional point was identified under fluoroscopy 3-4 cm laterally from the thoracic vertebrae on the side of the intervention. An extended indicator was used to reduce X-ray exposure of the surgeon. Then, a local anesthetic, prilocaine 60 mg, was injected into the subcutaneous tissues. The C-arm was placed for fluoroscopic guidance to obtain a lateral view, and the 18 gauge-3.5 inch needle was inserted into the middle of the disc. Then, the position of the needle within the disc was checked via AP and lateral views. Before ablation, the safety of the procedure was verified by applying negative motor nerve stimulation with short bursts to check the intradiscal location and the proximity of the L’DISQ^TM^ electrode to the nerve root. Close monitoring of pain is necessary to prevent thermal injuries. In addition, if the electrical stimulus causes lower extremity stimulation, the tip of the rod must be flattened and moved in an open position. In all stages, we continuously rotated the tip of the L’DISQ^TM^ electrode and moved it back and forth to increase ablation volume. Finally, the needle was pulled out, and a sterile bandage was placed to cover and dress the site; no sutures were used. No patient had neural damage or irritation after the procedure.

### Measurements of pain and satisfaction

The pain visual analog scale is self-completed by the respondent. Using a ruler, the score is determined by measuring the distance (mm) on the 10 cm line between the “no pain” anchor and the patient’s mark, providing a range of scores from 0-100: no pain (0-4 mm), mild pain (5-44 mm), moderate pain (45-74 mm), and severe pain (75-100 mm) (17).

Patients were asked the following four questions to determine their level of satisfaction 12 months after the procedure (North American Spine Society).

1. Very good: The procedure met my expectations.

2. Good:  I didn’t get as much improvement as I expected, but I can do the same for the same result.

3. Moderate: I didn’t get as much improvement as I expected, but I wouldn’t do the same for the same result.

4. Bad: I am the same or worse than before.

All patients were evaluated before and 1, 3, 6, and 12 months after treatment using the visual analog scale score. The Patient Satisfaction Scale was used to evaluate the level of patient satisfaction at the end of the treatment at 12 months.

Patients were permitted extensive walking, standing, and sitting down. They were instructed to avoid heavy lifting, forward skin bending or crushing. After 10-14 days, lightly working and home exercise with gentle flexion and extension were allowed.

### Statistical analysis

Data analysis was performed by ‘SPSS for Windows 21’ package program. Descriptive statistics were expressed as the mean ± standard deviation for variables with normal distribution, median (minimum-maximum) for variables with non-normal distribution, and number of cases and percentage (%) for nominal variables. The difference between the distributions of visual analog scale scores measured at different times was evaluated using the Friedman test. The Bonferroni Corrected Wilcoxon Signed-Rank test was used for multiple comparisons. For pairwise comparisons, the adjusted type 1 error level was accepted as 0/0.05 (0.05/10).

The power for non-parametric tests could not be calculated (18). Since the greater the magnitude of the effect, the greater the power, the clinical significance of this relationship was assessed using Kendall’s W correlation coefficient for Friedman’s ANOVA (18). Kendall’s W correlation coefficient was interpreted using Cohen’s guidelines of 0.1 (small effect), 0.3 (medium effect), and above 0.5 as a large effect (19). A p value of <0.05 was considered statistically significant.

## RESULTS

Eleven patients, 7 males and 4 females, were included in the study and were aged between 35 and 65 years. Nine patients had middle thoracic axial, and 2 had radicular pain whereas 4 patients had thoracolumbar disc degeneration and/or 7 had disc herniations at T10-T11 and T11-T12 levels, 8 patients had middle lumbar axial pain, and 3 had mild radiculopathy (Table 2). None of the patients previously underwent thoracic vertebral surgery.

Using the Friedman test, the difference between visual analog scale scores at the beginning (7.00±0.45) and the 1^st^, 3^rd^, 6^th^, 12^th^ months (3.55±0.69, 3.36±0.67, 2.55±0.69, and 2.73±0.65, respectively) were statistically significant (p<0.001). The results of pairwise comparisons using the Bonferroni Corrected Wilcoxon Signed-Rank test showed that there were statistically significant differences between the scores at the beginning and the 1^st^, 3^rd^, 6^th^, and 12^th^ months (p=0.003, p=0.002, p=0.003, and p=0.003, respectively). The mean visual analog scale score at the beginning (7.00±0.45) was significantly higher than the mean score of other months (Table 3).

Kendall’s W value was calculated as 0.759 for the visual analog scale score. It has a great magnitude of effect according to Cohen’s criteria because 0.759 is greater than 0.5. This result is clinically significant.

**Figure 3:**
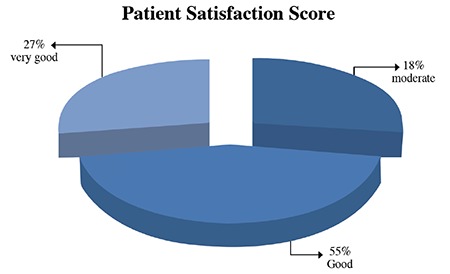
Patient satisfaction score.

When evaluated by patient satisfaction score at 12 months, 3 patients selected very good, 6 patients selected good, and 2 patients selected moderate. Overall, 82% rated the procedure as very good or good, and no patient rated it as worse (Figure 3).

## DISCUSSION

Initially, our patients’ visual analog scale scores were 7.00±0.45 points and then they were 2.73±0.65 points at 12 months. When compared with initial values, the visual analog scale scores of patients significantly decreased by 85%. According to the HMS survey, we achieved a value of 82% patient satisfaction. No complications occurred, and all patients were discharged the same day as the intervention.

Haufe et al. (11) reported 10 patients with thoracic disc herniation and/or degeneration who had percutaneous laser decompression and nucleotomy (PLDN) performed and were followed-up with visual analog scale scores. The median visual analog scale score was 8.5 points initially and 3.8 points after treatment. They used the PLDN technique in a patient group similar to ours, but they did not separate degenerative and herniated discs (11).

Hellinger et al. (12) performed PLDN in 42 patients with thoracic disc herniation. The majority of them had radicular and medullary pain. Satisfaction and success rate was reported as 90%. Forty-one reported an improvement six weeks later (14). Unlike our study, they performed interventions at all thoracic levels: C7/T1, T12/L1, and used a PLDN device. They reported the following three adverse events in 3 patients as pneumothorax, pleurisy, and spondylodiscitis. Their follow-up period was too short, and no long-term outcomes were reported. The results beyond the sixth week remain unknown. Our work included patients with radiculopathy while excluding those with root compression.

Lee et al. (20) evaluated data of patients with both radicular and axial pain (18) and with discogenic waist pain (21) who had lumbar disc decompression performed using L’DISQ^TM^. Kim et al. (22) studied patients with cervical herniated nucleus pulposus who were resistant to prior treatments. They concluded that the technique was effective in the cervical region as well as the lumbar region.

We suggest that the correct approach to reach the target level with proper control of the L’DISQ^TM^ electrode tip is effective for a successful outcome. In addition, therapeutic efficacy is significantly related to both patient selection and practitioner experience in all similar procedures. In conclusion, we propose that percutaneous ablation decompression L’DISQ^TM^ treatment may have an analgesic effect in select patients with thoracic disc herniation and discogenic/radicular pain.

## Figures and Tables

**Table 1:**
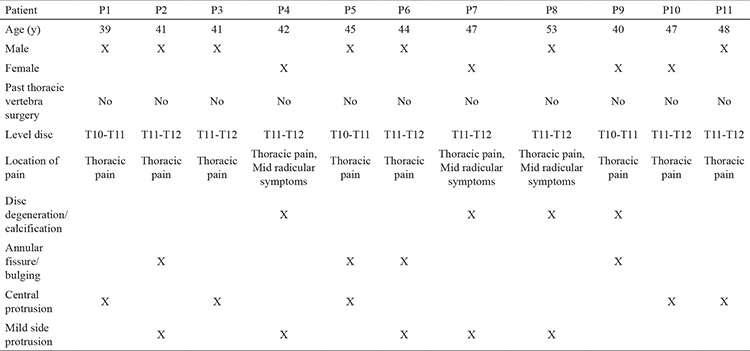
Patient characteristics and magnetic resonance imaging findings

**Table 2:**
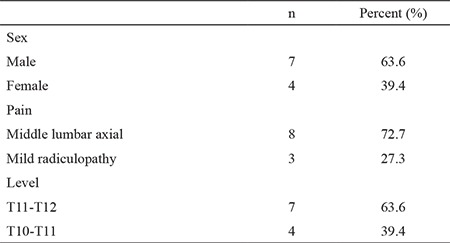
Demographic data

**Table 3:**
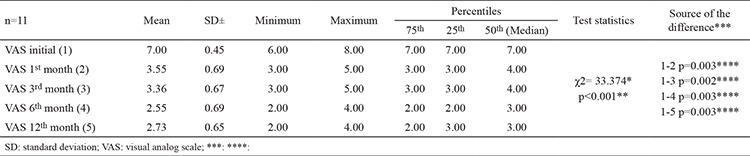
VAS scores according to month

**Figure 2:**
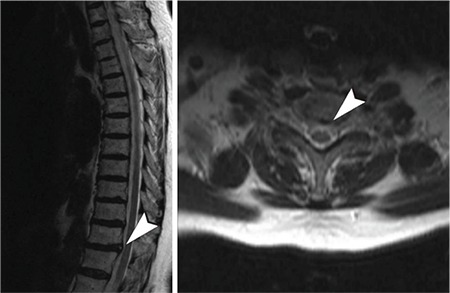
T10-T11 annular fissure, central protruded disc herniation.
